# Community perspectives on barriers and challenges to HIV pre-exposure prophylaxis access by men who have sex with men and female sex workers access in Nigeria

**DOI:** 10.1186/s12889-020-8195-x

**Published:** 2020-01-15

**Authors:** G. Emmanuel, M. Folayan, G. Undelikwe, B. Ochonye, T. Jayeoba, A. Yusuf, B. Aiwonodagbon, C. Bilali, P. Umoh, K. Ojemeiri, A. Kalaiwo

**Affiliations:** 1 Heartland Alliance International, Chicago, Nigeria; 2 New HIV Vaccine and Microbicide Advocacy Society, Lagos, Nigeria; 3 Department of Child Dental Health, Obafemi Awolowo University, Ife, Nigeria; 4 Joint United Nations Programme on HIV/AIDS, Lagos, Nigeria; 5 Heartland Alliance International, Lagos, Nigeria; 6 United States Agency for International Development, Abuja, Nigeria

**Keywords:** Men who have sex with men, Female sex workers, Pre-exposure prophylaxis, Nigeria, Sexually transmitted disease, Human immunodeficiency virus, Antiretrovirals

## Abstract

**Background:**

Men who have sex with men (MSM), female sex workers (FSW) have critical needs for effective HIV prevention tools. This study identified perspectives of MSM, FSW and policy makers on the needs for, barriers to, and challenges with pre-exposure HIV prophylaxis (PrEP); and the logistics required to support roll-out of PrEP for MSM and FSW in Nigeria.

**Methods:**

Qualitative and quantitative data were collected through a cross-sectional study. The quantitative data were collected through an online survey administered to 519 MSM, FSW and transgender respondents. The qualitative data were collected through 22 focus group discussions with 140 MSM and 80 FSW, and a two-day consultative workshop with 65 participants. Two open-ended questions in the online survey were also a source of qualitative data. Results of the quantitative data were reported descriptively; the qualitative data were inductively examined with a content analytic approach to construct descriptive categories. The findings from the quantitative and qualitative responses were triangulated.

**Results:**

Four hundred and ninety-four (95.2%) online respondents had heard about PrEP through community dialogue (71.3%), and 439 (84.6%) supported its use by MSM and FSW. Fewer than half of the respondents were aware of the clinical care required for PrEP, and misconceptions about PrEP were common. Stated barriers to PrEP uptake were stigma, cost, frequency of HIV counseling and treatment services required, and possible drug-drug interactions. Concerns included possible condom migration, increased risk for sexually transmitted infections and pregnancy for FSW, and poor adherence to medication and hospital schedules. Participants felt that trained peer educators and HIV-test counselors could provide information and refer clients to clinics that provide PrEP. PrEP can be provided through peer-led facilities for MSM and FSW, though its access should be expanded to all persons who are at substantial risk for HIV to prevent negative labeling of PrEP. Public awareness about the use of antiretrovirals for HIV prevention is needed to prevent labeling of PrEP users as being HIV positive.

**Conclusion:**

Although MSM and FSW are interested in the use of PrEP, numerous individual and structural barriers need to be addressed to facilitate access to it in Nigeria.

## Background

The prevalence of HIV infection among men who have sex with men (MSM) and female sex workers (FSW) in Nigeria is higher than the national HIV prevalence. At the end of 2013, the HIV prevalence was 19.4% among brothel-based FSW, 8.6% among non-brothel-based FSW, and 22.9% among MSM [1]. The high HIV infection prevalence in these populations increases the risk of transmission of HIV to the general community through their sexual partners, who act as bridges [2, 3]. For MSM, the risk of increasing infection within the community is also high since few community members are aware of their HIV-positive status, and only a few of those who know their HIV status receive treatment [4].

Multiple studies have highlighted the positive impact that the use of pre-exposure prophylaxis (PrEP) by persons at high risk for HIV infection has on the incidence of HIV [5, 6] in communities where the incidence of HIV infection is high [5]. The results of demonstration studies, such as the PROUD study and others, showed that it was feasible to offer PrEP daily to MSM; its use is safe, and consistent use has reduced the risk of HIV infection [7–9]. In FSW, similar demonstration studies on PrEP access found it feasible to offer PrEP daily to FSW, although retention in care can be problematic because of the migratory nature of sex work [10].

In 2015, Nigeria reported a study on the effectiveness of three PrEP delivery models for HIV-1 in sero-discordant couples [11]. The study was formulated by the outcome of research that identified the need to prioritise access of PrEP to those in HIV sero-discordant relationships [12] and to expand PrEP access to FSW and MSM [13]. There is, however, no roadmap on how to translate the results of PrEP demonstration project to actions and how to facilitate PrEP access by persons at substantial risk of HIV infection. The importance of a national roadmap for PrEP access was highlighted in the 2014–2015 National HIV Prevention Plan [14], the 2016 national HIV treatment guidelines [15], the 2018–2021 National HIV Prevention Plan, and the 2017–2021 National HIV and AIDS Strategic plan.

Despite national interest in facilitating access of people who are at substantial risk of contracting HIV infection to PrEP, the legal environment in Nigeria makes it challenging for MSM and FSW to access HIV prevention tools. The Same-sex Marriage Prohibition Act prohibits associations between MSM and other persons; others associating with MSM risk being jailed without the option of fines [16]. Thus, the law makes healthcare professionals uneasy about providing services to MSM. MSM are also less willing to identify and access services appropriate to their needs [17], and FSW face police harassment and are incriminated for possession of condoms, just as in the United States [18–20]. Social support for MSM also is poor [21]. These barriers raise concerns about how MSM, FSW and persons at substantial risk for HIV infection in Nigeria can access PrEP.

The aim of this study therefore was to identify perspectives that MSM, FSW and policy makers have on the needs for, the barriers to, and challenges faced with access to PrEP in Nigeria. The study also addresses the logistics required to support roll-out of PrEP to MSM and FSW. The outcome of the dialogue was to inform the scale up of PrEP access to MSM and FSW once the results of the PrEP demonstration project for HIV-1 sero-discordant couples in Nigeria were announced and the national PrEP roadmap is being developed.

## Methods

### Study design

This was a cross-sectional study that used quantitative and qualitative methods to generate information on perspectives of MSM and FSW in Nigeria regarding barriers to and challenges with access of MSM and FSW to PrEP. The quantitative data were collected through an online survey; the qualitative data were collected through focus group discussions (FGD) and a consultative meeting.

The study adapted the conceptual model by Ryvicker [22] on healthcare access, which had adopted a behaviour-ecological perspective. The model acknowledges that many factors affect healthcare consumers’ access to the healthcare system. The factors include availability of services, affordability, and transportation, as well as consumers’ health literacy, skills in communicating with healthcare providers, and social support to facilitate decision-making, adherence to treatment, and healthy behaviour.

### Online survey

The survey was a rapid assessment conducted to determine the extent of knowledge that FSW and MSM in Nigeria have of PrEP. The questionnaire for the online survey was adapted from the questionnaire used for the online survey of the Nigeria PrEP formative research [12]. It included two open- and 10 close-ended questions. Respondents were asked if they had ever heard of PrEP before reading the background information; how they learnt about PrEP; and if they perceived that PrEP had the potential to improve the national HIV response. They were asked also about their perception of potential barriers to PrEP by MSM and FSW They were also asked the reasons for their responses. Table 1 is a summary of the 10 questions asked in the survey.

**Table 1:** Result of online survey on perspectives about PrEP by FSW and MSM in Nigeria (*N* = 494)

Questions	No of respondents who agreed with statement	Percentage
It is a tool used for the prevention of HIV infection	339	68.6
Only HIV negative persons can take PrEP	295	59.7
Those at high risk for HIV would be given priority access to PrEP	233	47.2
Individuals on PrEP will have to take a pill about the same time each day	198	40.1
Individuals on PrEP will need to refill their pill supply regularly at a provider	159	32.2
Individuals on PrEP will need to get regular HIV tests	206	41.7
Individuals on PrEP will need to give blood samples regularly so that providers can monitor the effect of the pills on their organ	134	27.1
Individuals on PrEP will need to receive regular counseling to help them take their pills every day	180	36.4
Individuals on PrEP will need to receive regular counseling about how to reduce risk for HIV infection through safer sex behaviours	209	42.3
Pregnancy tests may always be required for female	104	21.1

The online survey was launched on June 28, 2016 and was open for 2 weeks. The survey participants had to be 18 years of age and to consent to participate in the study before they could respond to the survey. Also, persons interested in participating in the survey had to exclude themselves from research participation based on the listed criteria. The questions were preceded by an information page on PrEP -- what PrEP is, outcomes of the PrEP efficacy studies, and an update on ongoing PrEP demonstration studies around the world.

Information about the survey was shared with 30 key-population, community-based organisations in 14 states in Nigeria serving the needs of MSM and FSW. The organisations were encouraged to share the survey with their peers in the country.

### Focus group discussions (FGD)

Twenty-two FGD were conducted. 14 focus group discussions were conducted with MSM in Lagos, Akwa Ibom, Benue, Nasarawa, Kaduna, Cross River, Anambra, Oyo, Enugu, Kano, Rivers States and the Federal Capital Territory. Eight focus group discussions were conducted with FSW in Lagos, Akwa Ibom, Benue, Nasarawa, Kaduna, Cross River, Anambra in Oyo States.

The FGD explored information about appropriate target populations for PrEP, logistical barriers to PrEP access, possible facilitators of PrEP access, and requisites for using PrEP. The FGDs used case scenarios to elicit discussion, in which participants were given the opportunity to ask clarifying questions. Refreshments were provided during the FGD, and participants were compensated with $12.50 each as transport reimbursement. Immediately after each FGD, the facilitator wrote a summary report of the discussion.

### Consultative workshops

The meeting was convened on July 12–13, 2016 to discuss the findings from the online survey and the FGDs and to propose ways of facilitating access of MSM and FSW to PrEP in Nigeria. The meeting was conducted in English. The objective of the meeting was to identify barriers, challenges, and facilitators for PrEP for MSM and FSW; to develop strategies to address each barrier and challenge; and to develop strategies for educating MSM and FSW about daily PrEP. Detailed reports of the meeting proceedings were captured by two note takers. Various techniques were used to conduct the consultative meeting.

The meeting started with plenary presentations that informed participants of global, regional and national status on PrEP research and development. The aim of the presentations was to ensure that all participants had a common understanding on the issues about PrEP to be discussed at the meeting. Then, a roundtable discussion facilitated dialogues on policy and programmatic (and, and possibly community) responses to facilitate access to PrEP. Finally, two group discussions addressed knowledge gaps, roll-out strategies, creation of demand, and cost and funding for PrEP. One group consisted of community representatives and members of the non-governmental organisations. The second group consisted of policy makers, international partners, representatives of academia, and members of the National HIV Prevention Technical Working Group.

Group discussions were presented at plenary sessions for further brainstorming to generate new and creative ideas. Opportunities were provided for all participants to share ideas and for individuals to share stories that could strengthen their contributions to the discussion. An expert PrEP advocate facilitated the plenary discussion. The facilitation strengthened constructive discussions and consensus building through the collection, organization and summarization of the ideas generated from group discussions, using the nominal group technique [23]. The summarized findings were shared, and group conclusions and recommendations were generated.

### Data analyses

Descriptive analyses were conducted to determine the proportion of participants who responded to the questions. The data were automatically summarized and compiled with the Google survey tool. All non-respondents were categorized as negative responses.

The qualitative data consisted of the transcripts and summary notes from the FGDs, comments to the online questions, and the report of the consultative meeting. The analytic process began with a complete reading of the transcripts to identify content relating to the study objectives. Two designated team members worked in collaboration with a qualitative data analyst to develop a codebook and guidelines suitable for use with Atlas.ti. A selected set of texts was double-coded by two analysts to establish intra-coder and inter-coder reliability – a quality control measure that was automatically generated by Atlas.ti. After this quality check was completed, the coding team discussed coding discrepancies and resolved them by consensus during face-to-face sessions. The analysis continued until inter-coder reliability was at least 80%. After regular discussions were held among all the analysts to achieve standardisation and reliability, one of the analysts coded the remaining text. Themes for each of the questions were identified, and a streamlined list of quotes was developed.

### Study participants’ protection

The study received ethics approval from the Institute of Public Health Research Ethics Committee. All staff engaged in this study -- researchers and field workers -- received training on research ethics, with emphasis on the importance of confidentiality. No names or personal identifiers were recorded on any study instrument. All participants recruited for the research were 18 years and above. Written consent was obtained from study participants. The research team also felt it was important to exclude information of participants’ risk group from the survey to further heighten security for survey participants. All study-related information was stored securely and centrally at Heartland Alliance secretariat.

## Results

### Characteristics of study participants

Five hundred and nineteen responses were received for the online survey. Responses were received from 202 (41.3%) males, 284 (58.1%) females, 3 (0.6%) transgenders, and 30 (5.8%) who did not identify their sex. Five hundred eight (97.9%) respondents indicated their age. Of these, 19 (3.7%) were 10–19 years old, 185 (36.4%) were 20–24 years old, 233 (45.9%) were 25–34 years old, 65 (12.8%) were 35–44 years old, and 6 (1.2%) were 45–54 years old. No respondent was older than 54 years.

The FGDs consisted of 140 MSM and 80 FSW. The age range was 18–32 years. All participants had or were currently accessing peer education services through non-governmental organisations working with key populations.

There were 65 (38 males and 27 females) participants in the consultative meeting. Discussants included MSM and FSW from 12 states of the federation and the Federal Capital Territory. These states were the 12 states were the study was conducted and are listed among the identified 26 HIV intervention priority states in Nigeria [24]. Discussants also included representatives of eight donor agencies that fund the national HIV response and key population activities in Nigeria; five implementing partners that address key population issues in Nigeria; 25 non-governmental organisations with a national scope working on key population issues in Nigeria; academics; and members of the National HIV Prevention Technical Working Group.

### Interest, perception and knowledge about PrEP

Four hundred and ninety-four (95.2%) of the 519 respondents had heard about PrEP before the online survey; 453 (87.3%) supported the use of PrEP as a national HIV prevention tool; and 439 (84.6%) supported the use of PrEP as a HIV prevention tool for MSM and FSW.

Table 1 highlights the respondents’ perception of PrEP. A majority (68.6%) knew it is a tool used for prevention of HIV infection, and 295 (59.7%) knew that only HIV-negative individuals can take PrEP. Less than half of the respondents were aware of the clinical care that is required for PrEP uptake; only 206 (41.7%) knew PrEP requires regular HIV tests; 134 (27.1%) knew that PrEP requires regular blood draws to monitor the effect of PrEP on the kidney; and 104 (21.1%) knew that females must take regular pregnancy tests when taking PrEP. PrEP and post exposure prophylaxis were sometimes confused. One respondent to the online survey noted:

*“I just got to know that PrEP is treatment that is given to victims of rape to prevent them from being HIV positive because they cannot tell if the person that raped them is HIV negative or positive” – Respondent 10.*

### Barriers to PrEP access by MSM and FSW

Table 2 highlights possible barriers to PrEP access by MSM and FSW that respondents to the online survey identified. The main barrier was stigma and discrimination, identified by 296 (60.7%) respondents. Discussants at the FGD identified how stigma and discrimination may limit the uptake and use of PrEP. One of the reasons proferred was that use of an antiretrovirals implies being HIV infected. This misconception may result from poor public awareness that antiretrovirals are now being used for the prevention of HIV infection as well as for treatment of infection. Second, stigma and discrimination could result from drugs used for PrEP being labeled as drugs “for use by people with high HIV sexual risk behaviors.” Concern was raised by female sex workers on the use of emtricitabine Truvada® with a color similar to the drug use for HIV infection. A respondent during the focus group discussion noted.

**Table 2:** Result of online survey on barriers to PrEP access for MSM and FSW in Nigeria (494)

Questions	No of respondents who agreed with statement	Percentage
Lack of access to HIV testing and counseling	190	38.5
Affordability of regular health monitoring	226	45.7
Cost of PrEP to the individual	264	53.4
Stigma and discrimination	296	59.9
Using drugs or medicines that have unknown or toxic interactions with ARV	100	20.2
Costs of PrEP whe priority for resource allocation is treatment scale up	132	26.7

*“Clients may raise concern when they see me carrying with me drugs that looks like antiretroviral. This may be a source of violence for female sex workers.” – Focus Group Discussant, FSW*

Another barrier to PrEP identified by online survey respondents was cost -- cost of drugs; transportation costs; costs associated with regular monitoring of drug use; and costs of regular HIV counseling and treatment services – which were raised by 190 (38.9%). A fifth (20.2%) of respondents raised concerns about possible drug-drug interactions. A respondent was concerned about the government’s ability to provide PrEP, while another was concerned about increased risk for unwanted pregnancy.

*“The Nigeria government may not be able to provide PrEP for key affected population and even the general population.” – Respondent 22*

*“With the introduction of PrEP, I think the rate of abortion and unwanted pregnancy will increase.” – Respondent 160*

Concerns raised by focus group discussants were those of potential for side effects with the continued use of PrEP as a medication, the challenge of adherence, and increased risk of unprotected sex and sexually transmitted infection.

### Challenges with PrEP use by MSM and FSW

Challenges identified by focus groups for PrEP use were: condom migration because many individuals do not use condoms consistently; default of hospital visits due to poor perception of HIV risk; and the distance to reach PrEP clinics. Other factors that can pose a challenge are the cost of medication; frequency of monitoring required; long queuing time in public health facilities; and the high mobility nature of MSM and FSW lifestyle. These challenges can result in high rates of default at hospitals and clinics.

*“Female sex workers are highly mobile. Will the drugs be available and readily accessible if you need to get it anywhere else?” – Respondent 65*

Other challenges of PrEP identified by focus group discussants was the low priority that MSM and FSWs place on health care and community members’ low awareness of PrEP. These factors may on the one hand increase the risk for missed appointments and on the other hand limit the demand for PrEP. Other challenges that may play a role in poor participation of MSM and FSW in PrEP are unfriendly environments they encounter, especially in public health facilities, such as workers’ disrespect of them, inconvenient operating hours, and stock-out of drugs.

### Facilitators of PrEP uptake by MSM and FSW

The main way highlighted to facilitate PrEP uptake by MSM and FSW is education of community members. Table 3 reinforces this, as it relates that community dialogue is the main source of information on PrEP that respondents receive. Focus group discussants reported that many structures exist that can facilitate education of MSM and FSW about PrEP. One is the training curriculum and educational materials used for MSM and FSW peer-education sessions. Information about PrEP also can be shared at the drop-in-centres where FSW and MSM regularly access HIV counselling and testing services. Other ways identified by the focus group discussants is education about PrEP at brothels, bunks, clubs, special events organized to mobilize community members, outreaches, daily peer interactions, and the social media. Education should also be conducted by peer educators, healthcare workers, and brothel chair ladies, with their education integrated into community education programs conducted by community-based organizations working with key populations.

**Table 3:** Result of online survey on sources of information on PrEP for MSM and FSW in Nigeria (494)

Questions	Noumber of respondents who agreed with statement	*Percentage
Conference	85	17.2
Newsletter	44	8.9
Community dialogue	352	71.3
Online/Listserv	16	3.2
Newspaper publication	31	6.3
Others	16	3.2

*multiple responses possible

In addition to education, focus group discussants identified that referrals of FSW and MSM who test HIV negative at drop-in-centres where they regularly access HIV counselling and testing services would be helpful. However, HIV-testing counsellors and other health care professionals working at these drop-in-centres will need to be trained to communicate about the need for and use of PrEP.

### Suggestions for PrEP roll-out to MSM and FSW

Discussants at the national consultative meeting noted that PrEP access should be led by national and state governments. The national HIV and AIDS strategic plan should include PrEP access as one of the process indicators for HIV prevention for key populations in Nigeria. PrEP should also be included in the minimum prevention package of interventions for key populations and in all national policies and guidelines for access to HIV prevention commodities. Specifically, the national guidelines on use of antiretroviral drugs in Nigeria, the national HIV prevention strategic plan, and guidelines on HIV prevention for MSM and FSW should include details on PrEP access.

#### Strengthening of PrEP delivery models and health systems

Discussants at the national consultative meeting noted that PrEP should be delivered through public health facilities, private health care facilities and one-stop-shop HIV care centres for MSM and FSW. Moreover, PrEP should be provided free. PrEP access implies that the number of persons who visit the health care facilities will increase as clients who are not ill must visit the hospital. PrEP-access facilities will have to be strengthened to cope with the increased number of clients in need of PrEP who may visit the facilities.

Discussants identified that MSM and FSW access health care services from non-health sector personnel. Access of PrEP through the non-health sector needs to be included in the model of PrEP delivery in order not to leave a group of MSM and FSW behind. Whatever the model of PrEP delivery is, the community should drive demand, and PrEP delivery should be integrated with access to other HIV prevention services, and be provided through a one-stop-shop model.

#### Capacity building

Discussants at the national consultative meeting noted that the number of skilled and unskilled health care providers should be increased to facilitate access of community members to PrEP through an integrated health care delivery system. Training is required on how to identify and refer HIV-negative individuals at substantial risk for HIV for PrEP access. Referral systems should to be strengthened, as persons who test either positive or negative to HIV should be referred. These training can be limited to the staff at clinics designated to provide PrEP. Those clinics should be supported to address Truvada® supply logistics, prevention of stock-outs, and PrEP adherence counselling for MSM and FSW.

#### Populations to be targeted for PrEP

Respondents to the online survey and discussants at the FGD stated that persons at substantial risk for HIV -- not only MSM and FSW -- should be eligible for PrEP. These populations should include other men and women in sexual relationships who may be at substantial risk for HIV infection such as HIV sero-discordant couples, adolescents and young women and widows. One of the respondents on the online survey noted that:

*Yes those that are considered to be at substantial risks [for HIV infection] are adolescents girls & young women, female sex workers, men [who have] sex [with] men, sero-discordant couples – Respondent 27.*

#### Platform for knowledge sharing

Discussants at the national consultative meeting noted that a national platform for sharing knowledge and best practices on PrEP access and use should be created. This platform could be integrated into a national HIV conference or part of a platform post exposure prophylaxis to specifically address HIV prevention in Nigeria.

## Discussion

This study reveals that a significant proportion of MSM and FSW in Nigeria are aware of and interested in PrEP. However, they have limited understanding of the clinical care required for PrEP, and they confuse PrEP and post exposure prophylaxis. Study participants identified stigma as barriers to PrEP use. The barriers include the implication that use of antiretroviral equates with being HIV positive and the labelling of PrEP products as “for persons at substantial risk for HIV infection.” Increases in risk of unwanted pregnancy and sexually transmitted infection were also concerns expressed about PrEP. Challenges to wider use of PrEP may be the numerous scheduled hospital visits required, inefficient delivery of care in public health facilities, and the high mobility of FSW. Existing systems and structures, such as peer educators and peer-led one-stop-shop clinics accessible to MSM and FSW, might be used to increase awareness, address misconceptions, and facilitate access of MSM and FSW to PrEP. Peer educators and health care providers will need to be trained on how to educate MSM and FSW about PrEP and to refer for those who are HIV negative yet are at substantial risk for HIV infection for PrEP. Access to PrEP should be made available for all persons at substantial risk for HIV infection and should include reducing the stigma associated with prophylaxis and the perceived inappropriate labelling of PrEP products.

A strength of this study was its harnessing views and perspective of diverse and critical stakeholders involved with the HIV response for MSM and FSW in Nigeria. While the online survey reached out MSM and FSW who had access to the internet, the FGD reached out to those who may not have access to the survey, and the consultative meeting reached out to policy makers and important others, such academicians. Thus, the views and perspectives captured in this study are those of an array of critical stakeholders for PrEP delivery in Nigeria.

This study identified stigma as a major barrier to PrEP access. Attention should be paid to preventing stigmatisation of those who use PrEP, as stigma limits the uptake and use of PrEP just as stigma impeded the initial uptake of HIV treatment [25], contraception [26] and preventive treatment [24]. Though the reported proportion of people living with HIV who experienced stigma in health care settings decreased from about 20 to 14% from 2010 to 2012 [27], stigma still limits uptake of HIV treatment by those living with HIV [28].

A new form of stigma also arises with the use of PrEP, as highlighted by Calabrese and Underhill [29] and Haire et al. [30]. PrEP may be labelled as product used by sexually promiscuous individuals and associated with HIV, homosexuality and sex work [29]. This stigma impacted negatively on PrEP uptake in the United States [29, 31] and required intensive campaigning to increase public awareness and uptake by MSM [32]. Ensuring that PrEP is prescribed for persons at substantial risk for HIV infection, other than MSM and FSW such as adolescents, young women, and HIV sero-discordant couples, may reduce the reluctance of MSM and FSW to access PrEP. as the unfavourable legal environment has negative implications for labelling PrEP as product for use by MSM and FSW [18]. Ironically, the unfavourable legal environment was not identified as a limiting factor for PrEP access in the discussions.

The emphasis on public awareness about the use of antiretrovirals for prevention as well as treatment of HIV treatment, and preventing the stigmatisation of those who choose to use PrEP, highlights the seriousness of HIV-related stigma. The inability of the HIV field to curtail HIV-related stigma that people living with HIV face likely impedes success of the HIV prevention field. Idoko et al. [12] highlighted how HIV serodiscordant couples had found that picking up PrEP from HIV treatment clinics led to assumptions that those who are HIV negative have HIV infection, thus increasing their risk for stigma and discrimination. The broad scale use of antiretrovirals should facilitate integration of HIV treatment and HIV prevention into routine clinical care in general out-patient clinics, thus decreasing the labelling of those that attend antiretroviral clinics.

PrEP access through the public health facilities will, however, require investment in increasing capacity to care for the teeming number of hospital clients who are well. Hospitals may need to consider task-shifting to reduce the burden of PrEP provision on professional health care workers. Task-shifting of clinical activities can significantly increase clinic capacity to screen, follow-up and retain persons on PrEP [33]. These public health facilities will also need to be key population friendly to support MSM and FSW use of the facilities. PrEP uptake can be facilitated through peer-led services since MSM and FSW express satisfaction with those facilities [34]. Satisfaction with service provided to MSM and FSW in public and private hospitals can be improved through training of personnel [35]. While it may not be possible for all public and private health facilities to provide PrEP, it will be appropriate that all peer-led health facilities provide PrEP for MSM and FSW. Although the cost of PrEP access is less in public health facilities than in private facilities [33], there are no studies on the cost of PrEP provided to key populations through peer-led health care facilities; it is possible that care will be cheaper in those facilities since they serve as one-stop-shops for HIV services.

Cost is a significant barrier for PrEP access as it is for HIV treatment and other health-related commodities [36, 37]. In the United States, PrEP cost is subsidised through numerous schemes [38], while in France it is reimbursed through the health system [39]. PrEP is paid for by out-of-pocket expense in Canada, Australia and United Kingdom [40–42], although provinces’ funding to cover the drug cost is feasible in Canada [39]. Kenya and South Africa are the only African countries that support access of PrEP by communities at risk for HIV infection, which they do through donor-funded projects.

Other concerns raised in this study about PrEP -- behaviour disinhibition; increased risk for pregnancy, HIV transmission, and sexually transmitted infections; and toxicity of the medications -- have been raised also in other parts of the world [43–45]. Although no studies have shown increased sexual behaviour risk by people taking PrEP [46–50], the high prevalence of sexually transmitted infections observed in PrEP demonstration studies raised concerns. A recent meta-analysis, found an increase in risk for sexually transmitted infection with PrEP use [51]. This increase may actually be a reflection of PrEP uptake by those with high risk for HIV acquisition due to poor condom use rather than an increase in risk for HIV acquisition by those on PrEP [52]. Drug toxicity has remained low in PrEP studies [53–56].

Risk for pregnancy and abortion is however a real concern as raised by some discussants. The prevalence of unprotected sex by female sex workers in Nigeria is a real concern. Only 92.3% of FSW reported always used condom during sex in the last 30 days in Nigeria [57]. There are very few reports on pregnancy intention of FSW in low and middle income countries with the most of the few studies reporting high incidences thus representing a considerable concern [58]. Efforts need to be made to mitigate this risk for FSW who use PrEP as an alternative to consistent condom use.

One limitation with this study design is that it did not selectively analyse data from adolescent MSM and FSW. Thus, application of the results to this population is limited. Access of adolescents to PrEP can be challenging [59] despite this population being at high risk for HIV infection, even in Nigeria [60]. Unfortunately, little is known or understood about adolescent MSM in Nigeria, and this study’s findings may not be applicable to adolescent MSM and FSW. Also, the study could not disaggregate the qualitative data by HIV risk groups. The study team decided not to collect data on the group because of concerns about identification of respondents. Although we cannot rule out the possibility that individuals other than MSM and FSW responded to the online survey, we limited this possibility by sharing information about the survey only with the communities of MSM and FSW in Nigeria and requested that they share the information with their peers. Despite these limitations, the study findings provide information that can shape policies and programmes for PrEP delivery to MSM and FSW in Nigeria in the near future.

## Conclusion

Although a high proportion of MSM and FSW is interested in using PrEP for HIV prevention in Nigeria, many individual and structural barriers may need to be addressed to enhance successful implementation of any PrEP programme for the community. Addressing these challenges through the use of existing systems and structures rather than creating new structures will help ensure a cost-effective PrEP programme and reduce the delay in popularising PrEP by those most in need of it.

## Data Availability

The datasets for the study are available from the lead author on request. Though no identifying details are contained in the dataset, due to the homophobic and discriminatory environment in Nigeria towards MSM and FSW, the overall security of the MSM and FSW communities is of paramount interest.

## Abbreviations

ARV: Antiretrovirals
FGD: Focus Group Discussion
FSW: Female Sex Workers
HIV: Human Immunosuppression Virus
MSM: Men who have Sex with Men
PrEP: Pre-exposure Prophylaxis
